# Bohemian Knotweed *Reynoutria × bohemica* Chrtek et Chrtková Seems Not to Rely Heavily on Allelopathy for Its Persistence in Invaded Sites in the Southwest Part of the Zagreb, Croatia

**DOI:** 10.3390/plants12112222

**Published:** 2023-06-05

**Authors:** Damjana Levačić, Lucia Perković, Nina Vuković, Sven D. Jelaska

**Affiliations:** 1 Department of Biology, Faculty of Science, University of Zagreb, Rooseveltov Trg 6, 10000 Zagreb, Croatia; damjana.levacic@biol.pmf.hr (D.L.); nina.vukovic@biol.pmf.hr (N.V.); 2 Ekonerg—Institute of Energy and Environmental Protection, Koranska 5, 10000 Zagreb, Croatia; lucia.perkovic@ekonerg.hr

**Keywords:** allelopathy, Europe, *Fallopia*, germination test, invasive plants, Kruskal–Wallis ANOVA, soil chemical characteristics

## Abstract

Notorious invasive Bohemian knotweed *Reynoutria × bohemica* Chrtek et Chrtková is a hybrid of two species, *Reynoutria japonica* Houtt. and *Reynoutria sachalinensis* (F. S. Petrop.) Nakai in T. Mori which spontaneously developed in Europe, outside the natural distribution of its parental species. Its success could potentially lie in its allelopathic activity, which was confirmed in a number of experiments conducted with the leaf and root exudates, testing their effect on the germination and growth of various test plants. Here, we tested its allelopathic potential using different concentrations of leaf exudates on two test plants, *Triticum aestivum* L. and *Sinapis alba* L., in Petri dishes and pots with soil and by growing test plants in the soil sampled in knotweed stands on the edges of stands and outside of stands. Tests in Petri dishes and pots with soil to which leaf exudates were added have shown a decrease in germination and growth in comparison to the control, hence confirming the allelopathic effect. However, this was not confirmed in a test with in situ soil samples, where no statistically significant differences were observed, neither in the growth of test plants nor in the chemical characteristics (pH, soil organic matter, humus content) of the soil. Therefore, the persistence of Bohemian knotweed at already invaded sites could be attributed to its efficient use of resources (light and nutrients) through which it outcompetes native plants.

## 1. Introduction

As one of the biggest threats to biodiversity [1,2,3] and ecosystem processes [4,5], invasive alien plants (IAPs) often establish competitive superiority over native plant species [6,7] through distinctive morphological traits that directly improve fitness [6] and/or physiological traits that restrict other organisms’ germination, growth, development and reproduction [8].

Novel Weapon Hypotheses (NWH) [9] suggests that IAP increase their competitive ability through the possession of allelochemicals unencountered by native species [10]. In the original native range, such chemicals fail to exhibit toxic effects on other species because of the coevolved tolerance due to long-term association [11]. Allelopathy is an interference mechanism of living or dead plants conducted through volatilization, leaching, litter decomposition and root exudation [12,13,14] which releases allelochemicals into the air or soil exerting an effect (mostly negative) on co-occurring plants [15,16] or within a species (i.e., autotoxicity [17]. Allelochemicals can directly inhibit neighboring native plants by disturbing the photosynthesis, respiration, and metabolism systems, or indirectly suppress them via disruption of beneficial belowground microbial mutualists, or altered environmental conditions, including soil physicochemical properties [18,19]. Allelopathy is a ubiquitous mechanism of invasion widespread across the plant phylogeny [20], so the success of IAP attributed to allelochemicals has been debated. Moreover, the novelty of invader’s allelochemicals to native species diminishes over time [21].

Out of the 77 Croatia’s invasive alien plant taxa listed in the Flora Croatica Database [22] (accessed on 14 February 2023), all three taxa from the genus *Reynoutria* are invasive: *Reynoutria japonica* Houtt. (Japanese knotweed), *Reynoutria sachalinensis* (F. Schmidt) Nakai (giant knotweed), and their hybrid *Reynoutria* × *bohemica* Chrtek et Chrtková (Bohemian knotweed).

Taxa from the genus *Reynoutria* are robust herbaceous perennials belonging to Polygonaceae family with spring emerging above-ground stems developing from the over-wintering rhizome [23]. Today, it is well known that taxa of this genus possess many characteristics of an “ideal invader” including an allelopathic component [24]. Rapid clonal growth, which can reach up to 15 cm a day [25], efficient regeneration from perennial rhizomes [26] and an increased production of allelopathic metabolites in the non-native range [1,27] enable knotweeds to dominate the areas they have occupied. Previous research has suggested that aqueous exudates of *Reynoutria* leaves could reduce early growth of the seedlings [28]. Exuded allelochemicals may trigger a series of changes in soil pH, organic matter content, nutrient availability, mycorrhizal and microbial community diversity and abundance, posing a threat to native vegetation [14]. 

High and fast biomass production results in extremely dense stands that prevent light from reaching the ground level of vegetation, which, along with allelopathy, causes a general decrease in flora diversity in the area and the absence of certain plant species [29]. In addition, a number of studies indicate that the presence of knotweeds in the area has an impact on the surrounding fauna [30,31,32,33,34].

First knotweed to be mentioned in Croatia was *Reynoutria japonica* in the first half of the 20th century, although it was probably present even earlier [22]. Typical thriving habitats for knotweeds are mainly disturbed habitats along the river banks, roads or railways [35,36], which knotweeds occupy mostly in the lowlands of northwestern and eastern parts of the country. Because of their sprawling network of rhizomes, management and control measures are very time consuming, labor intensive and costly [37,38,39].

Since *Reynoutria* × *bohemica* spontaneously developed in a new area outside the natural range of its parent species, its existence was not recorded until 1983 [40], although present in Europe for much longer [41]. In Croatia, *R.* × *bohemica* was first recorded in 2015 [23], but a more detailed examination of herbarium specimens revealed that the parent species *R. japonica* was often wrongly determined. Today, it is considered that *R.* × *bohemica* is the most widespread species of the genus *Reynoutria* in Croatia [42]. 

With the abundance of invasive traits, it is considered that this hybrid spreads faster and more aggressively than its parents [43,44] primarily by forming dense and tall stands [29], while the extensive and regenerative rhizome system indicates its increased competitiveness for nutrients [45]. Dramatically reduced plant species richness in the vicinity of an *R.* × *bohemica* growing area, heavily modified plant communities [34], the lack of seedlings of other species in the early spring before the vegetation peak [46] along with the absence of shade-tolerant species indicate an allelopathic effect [1,29,47,48]. 

Allelopathy can be conditional with discrepancy between geographical sites, where the same species exhibit allelopathic invasion in some but not the other areas [49]. Environmental factors such as temperature, light, soil nutrients and microorganisms may strengthen or alleviate the allelochemical activity [50]. In some cases, leaf litter and leachates cannot solely show allelopathic potential, but only when united with other biotic or abiotic factors [51,52]. The content of allelochemicals can vary in different soil textures [52] and can be limited by soil moisture [53].

The aim of this study is to test the potential allelopathic effect of the invasive plant *Reynoutria* × *bohemica* Chrtek et Chrtková directly using leaf exudates and indirectly using soil from the natural stands on the germination and growth of white mustard (*Sinapis alba* L.) and common wheat (*Triticum aestivum* L.). We hypothesize that the allelochemicals from the leaf exudates and the soils affect the germination and growth of the test plants as well as cause changes in the chemical characteristics of the soil.

## 2. Results

### 2.1. Germination and Growth Test

Initial testing of the allelopathic potential of Bohemian knotweed leaf exudates conducted in Petri dishes has shown that white mustard is not affected (at 0.01 g/mL) or is mostly negligibly affected (at 0.03 g/mL and 0.05 g/mL), while common wheat is much more influenced, especially at the highest concentration where only one third of the seeds germinated (Table 1). Based on these results, for the test with pots with soil into which the leaf exudates were added, we used only the 0.05 g/mL solution from the initial testing in the Petri dishes and additional exudates made at a 0.1 g/mL concentration. Again, white mustard seeds were less affected than common wheat seeds, although differences were smaller when compared to the Petri dish test results (Table 1). In the pot experiment, the control had a lower germination success rate than the Petri dishes as well. This can be explained by the fact that the seeds in Petri dishes, as opposed to seeds in pots with soil, had easier access to the moisture required in the early stages of germination. Additionally, the Petri dishes test only lasted for five days, whereas the pot test lasted for fourteen, because we were also interested in the potential allelopathic effect on seedling growth. As a result, some of the seeds that initially germinated have not grown shoots and eventually died off. The latter could have an impact on the percentage of seeds that successfully germinate depending on whether they are counted as soon as the first root breaks or based on presence of developed seedlings.

**Figure 1:**
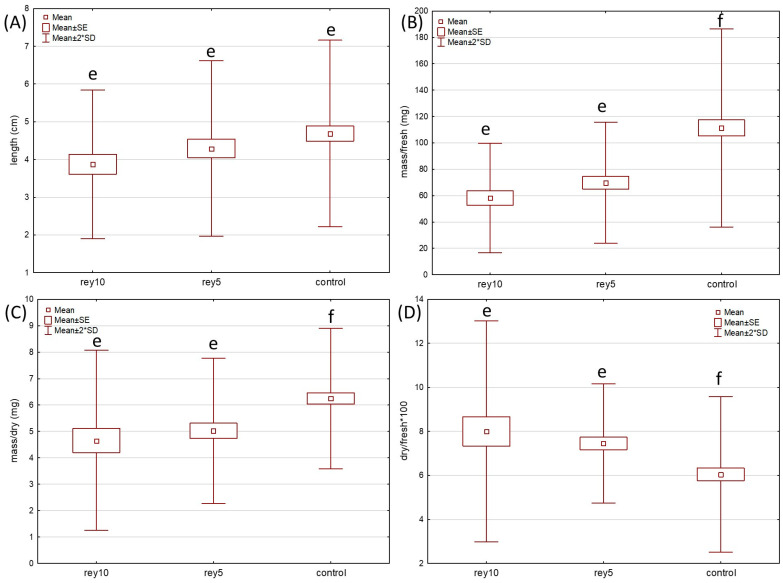
Growth parameters ((**A**)—length; (**B**)—fresh mass, (**C**)—dry mass, (**D**)—dry/fresh mass ratio) of *Sinapis alba* shoots grown in pots with soil with two treatments (watering with rey5–0.05 and rey10–0.1 g/mL of Bohemian knotweed leaf exudates) and control (distilled water). Statistically significant differences (*p* < 0.05) indicated with different letters.

**Figure 2:**
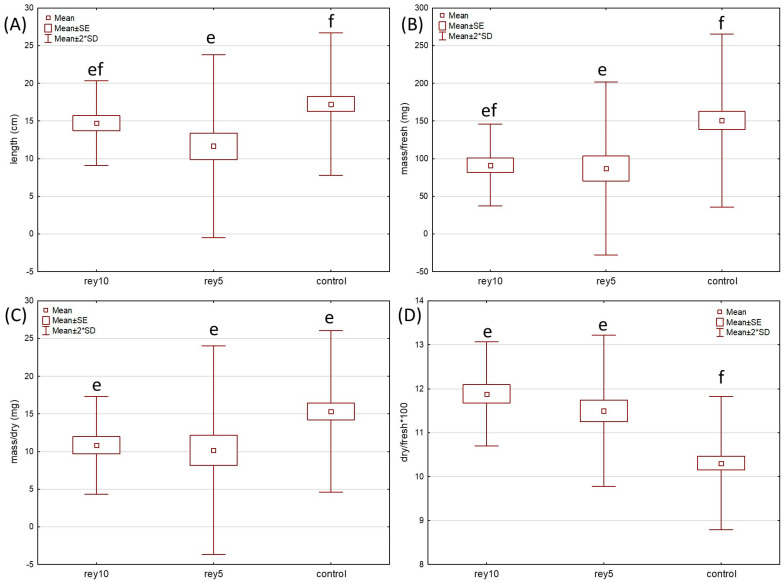
Growth parameters ((**A**)—length; (**B**)—fresh mass, (**C**)—dry mass, (**D**)—dry/fresh mass ratio) of *Triticum aestivum* shoots grown in pots with soil with two treatments (watering with rey5–0.05 and rey10–0.1 g/mL of Bohemian knotweed leaf exudates) and control (distilled water). Statistically significant differences (*p* < 0.05) indicated with different letters.

Kruskal–Wallis ANOVA and post hoc comparisons of mean ranks showed that there are some statistically significant differences in the four measured parameters of the shoots (fresh mass, dry mass, their ratio, and length—measurements in Tables S1 and S2 in the Supplementary Material) between treatments (watering with 0.05 and 0.10 g/mL of Bohemian knotweed leaf exudates and control with distilled water) in both test plants (Figure 1 and Figure 2). The Kruskal–Wallis test and post hoc comparisons of mean ranks (Tables S3–S6 in the Supplementary Material) revealed that for *Sinapis* shoot fresh mass, dry mass, and ratio of the former, there were statistically significant differences between control and both leaf exudate treatments, while there was no difference in shoot length. For *Triticum* shoots (Tables S7–S10 in the Supplementary Material), control was statistically significantly different from the 0.05 g/mL treatment for all the parameters except dry mass and from the 0.1 g/mL treatment for the dry/fresh mass ratio. The fact that length and fresh and dry mass of shoots of both test species had higher mean values at control seedlings confirms the potential allelopathic effect of Bohemian knotweed leaf exudates.

**Figure 3:**
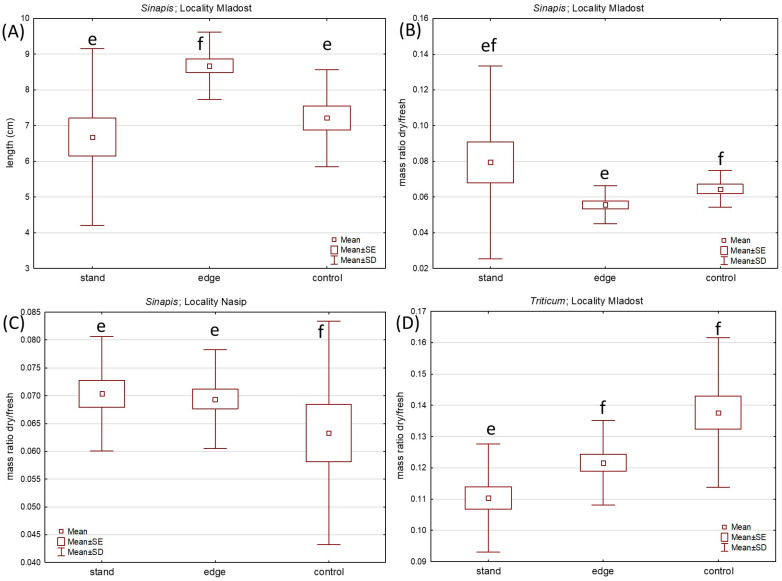
Growth parameters of *Sinapis alba* (**A**–**C**) and *Triticum aestivum* (**D**) shoots grown in pots with soil sampled in triplicates on each site, inside the stand, at the edge of the stand, and controls outside the stand of *R.* × *bohemica,* for which there were statistically significant differences (*p* < 0.05) indicated with different letters.

In the third stage of our Bohemian knotweed allelopathic potential research, we tried to mimic more realistic circumstances by growing test plants in pots with soil sampled in situ at the three knotweed-invaded sites. Given that all three sites had dense knotweed stands that had been continuously present for at least 10 years, we presumed that the soil should contain potential allelopathic compounds originating from the knotweed. Based on the four parameters of shoots (fresh mass, dry mass, their ratio, and length—measurements in Tables S11 and S12 in the Supplementary Material) between test plants grown in soil taken within the knotweed stands, at the edge of the stands, and outside of the stands as control, Kruskal–Wallis ANOVA and post hoc comparisons of mean ranks showed that there are just a few statistically significant differences in both test plants (Figure 3, Tables S13–S16 in the Supplementary Material). Observed differences do not reveal a clear pattern, which should point to differences with respect to the position of soil samples in relation to the knotweed stand. These findings do not prove that allelopathic substances, which may have a considerable detrimental effect on the growth of other plant species, are present in soil that has been impacted by the long-present knotweed.

### 2.2. Soil Chemical Characteristics Test

Comparison of three soil chemical parameters (Table 2) of the soil samples from the three Bohemian-knotweed-invaded sites collected simultaneously with those in the previous growth test yielded results similar to those in the latter. Kruskal–Wallis ANOVA test showed that there are no statistically significant differences with respect to the position of the soil samples in relation to the knotweed stands at all three locations. The only exception was the Mladost location, at which a statistically significant difference was found for pH values between soil sampled at the control and inside the knotweed stand (Table S17 in the Supplementary Material). Therefore, it appears that knotweed has little effect on the chemical characteristics of the soil.

## 3. Discussion

Allelopathy potential has been confirmed for numerous invasive plants. In their analyses of 524 invasive plant species belonging to 113 families, Kalisz et al. [20] determined that allelopathy occurred in 51.4% of plant species and is found in 72% of corresponding plant families, making it a pervasive trait among invasive species. However, whether and to what extent plant species employ its allelopathic potential is quite complex, and depends on multiple factors. Xu et al. [14] provided a detailed review on the topic, dealing with allelopathy in grasslands and forests, taking into account the chemical nature of the allelochemicals, plant–soil and plant–plant interactions. 

Here, we confirmed that the allelopathic potential of Bohemian knotweed leaves exudates on germination and growth of the two test species (Table 1, Figure 1 and Figure 2), while this was not confirmed when using in situ soil from the knotweed stands. Out of the 540 total sown seeds in the latter group, 389 germinated and developed into a plant, which makes a 72.03% germination and growth success rate. It should be noted that the control germination test of the test plants in Petri dishes with distilled water showed a 95% success rate, which verified that the seeds were healthy and suitable for conducting this experiment. The growth analysis of *S. alba* and *T. aestivum* yielded only a few statistically significant differences, without a clear pattern that could be attributed to soil sampling position (control, edge or inside the stand). Three (out of four) statistically significant differences were found for the dry/fresh mass ratio (Tables S14–S16 in the Supplementary Material). However, these differences observed at the same location (namely Mladost) were not identical between the two test plants. For *S. alba*, there is a difference between plants grown in soil sampled at control and the edge of the Bohemian knotweed stand, while for *T. aestivum*, difference was observed for plants grown in soil sampled inside the stand in comparison to other two samples. Generally, values of the measured parameters (fresh mass, dry mass, their ratio and length) did not indicate that *R.* × *bohemica* affected test plant’s growth. The same conclusion was reached by Mincheva et al. [54] who investigated the growth of native species—*Lolium perenne* L. and *Trifolium repens* L.—in soil previously invaded by Japanese knotweed. Another set of authors, Parepa et al. [55], also claim that plant litter and soil influenced by *R.* × *bohemica* do not have negative effects on germination, biomass and diversity of native species. 

On the contrary, Murrell et al. [1] propose that the effect of *R.* × *bohemica* on native Central European species contains a strong allelopathic component. The addition of activated carbon to the soil, which absorbs organic compounds, improved the growth of native species. This effect was similar to the effect of repeated removal of *R.* × *bohemica* shoots. Unlike in the case of some other invasive plants [56], damage to the aboveground parts does not induce increased allelopathy, so regular cutting is claimed to be a safe and ecologically risk-free way of keeping *R.* × *bohemica* under control, mitigating its ecological impact especially in the early stages of invasion. Vrchotova and Šera [57] claim that the exudates of the aboveground plant parts have a stronger inhibitory effect on *S. alba* than the rhizome exudate and that the exudates of yellow autumn and green summer leaves of the Japanese knotweed reduce its germination by 22%. This differs from our results, where the germination of white mustard was greater than 90% when treated with all concentrations of leaf exudates. Additional aspect of using different concentrations of plant parts exudates in allelopathy testing could be the different osmotic potential among treatments (i.e., different concentrations) and control (e.g., distilled water). This has been analyzed by Loydi et al. [58], who found that lower osmotic potential does negatively affect the germination percentage, mean germination time and root length of test plants, but also that the effects of non-native plants litter leachates on germination of test plants were more negative than the pure osmotic effects.

The obtained results did not confirm the initial hypothesis that the soil influenced by *Reynoutria* × *bohemica* would show significant changes in the analyzed chemical characteristics within each of the three sampled localities. The only exception was statistically significant difference in pH value of the soil sampled inside the Bohemian knotweed stand in comparison to the control, at the Mladost location. Overall, pH values (Table 2) proved to be similar in all three locations and sampling positions with values that classify them in the category of moderately alkaline soils. Dassonville et al. [59] studied parent species’ (*R. japonica*) influence on soil pH and showed somewhat lower values compared to those of this study, suggesting that the soil under invasion pressure makes a moderately acidic environment. In contrast, soil pH values obtained by Maurel et al. [60] displayed a slightly alkaline reaction, corresponding to the results of our study. None of these studies confirmed statistically significant change in the pH value of soils under the invasion pressure, suggesting that the presence of knotweed alone does not affect this change, but other environmental parameters are responsible for individual deviations in the results.

The highest concentrations of organic matter were recorded at the Mladost location—control position. It is important to note here that the Mladost location is situated at the forest edge, so the control samples were collected within the forest area, which could explain the increase in organic matter. As for the actual influence of the *R.* × *bohemica* on the amount of organic matter in the soil, based on the obtained results, it is negligible. Maurel et al. [60] state that the presence of *R. japonica* has a strong influence on the organic matter in the soil, which can be explained by extreme above-ground and underground biomass. According to Maerz et al. [32] and Dassonville [61], the parent *R. japonica* produces abundant plant litter that slowly decomposes, thus creating thick accumulations in the soil. According to Ehrenfeld [62], invasive plants can increase nutrient production in the occupied area, which helps them in further invasion by ensuring enough nutrients. As contents of humus and organic matter are closely related, the obtained values for humus in the soil follow a trend of change by locations and positions relatively similar to that of organic matter. 

Overall, these types of studies mostly examine only the influence of plant exudates from leaves or rhizomes, which we did in the first part of our research. Studies have shown that certain allelopathic compounds can be broken down in the soil, which reduces their allelopathic effect in relation to their allelopathic potential [63], and also that certain allelochemicals that knotweeds release into the soil do not persist long or are washed away into deeper layers [48]. Accordingly, Mincheva et al. [54] claim that after a certain time, soil that was once under the influence of Japanese knotweed loses the allelopathic properties that the knotweed could have potentially transferred, and because of this, the soil can recover relatively easily once the complete knotweed biomass is removed. 

The chemical nature of the allelopathic substances, different physicochemical properties of various types of the soil, as well as different soil biota can results in different pathways of the allelopathic substance decomposition as discussed by Xu et al. [14]. Furthermore, recent studies [64,65] have shown that production of the allelochemicals can be dependent on the neighboring plants’ identity, increasing it in the presence of competitor species/individuals. The latter could result in a decrease in the necessity of using resources for allelochemical production as the invasion process progresses, hence the decrease or even complete lack of other species in the neighborhood. Consequently, expression of plant allelopathic potential could be spatially and temporally dependent. Although this makes using in situ soil in tests of allelopathy quite complex, and not easy for yielding straightforward conclusions, we believe that these are necessary to obtain better perspective on the topic.

Bohemian knotweed creates exceptionally dense monocultures and an extensive rhizome system where native plants are almost completely suppressed [66], which puts competition on the pedestal for the main mechanism for the successful invasion. Our results obtained with in situ sampled soil support these claims, but since there are studies that confirm intense allelopathic effect of *R.* × *bohemica*, it could still be, along with a strong competitive ability, one of the components that further help its spread, especially in the early stages of the invasion process. It is important to emphasize the difference in conditions between the nature with multiple complex interactions and the laboratory where most of the research on allelopathy is carried out. Another obstacle in making the right conclusions could also be the use of agricultural or non-native weed test species instead of natives suppressed by invasive species in their natural habitat [9]. Here, we have used seeds of two species (i.e., mustard—*Sinapis alba* L. and wheat—*Triticum aestivum* L.) that mostly do not coexist with Bohemian knotweed in the invaded areas; hence, the obtained results could hinder yielding accurate general conclusions. Nevertheless, both test species are widely commercially available and frequently used in these types of testing, enhancing comparability of results from other authors. Furthermore, as representatives of two large and widely distributed plant families (i.e., *Brassicaceae* and *Poaceae*) whose members do coexist with the knotweeds in the invaded areas, mustard and wheat that share some traits within their respective families with knotweed-coexisting species, could provide results that might be informative and useful. 

In order for allelopathic research to be as representative as possible, it is necessary to conduct more investigations in the field or to mimic conditions as accurately as possible to those in the natural environment, along with using native species. An additional aspect of future research on the allelopathic potential of Bohemian knotweed could be in comparing the presence of this mechanism during different stages of the invasion process (introduction–establishment–dominance). 

## 4. Materials and Methods

**Figure 4:**
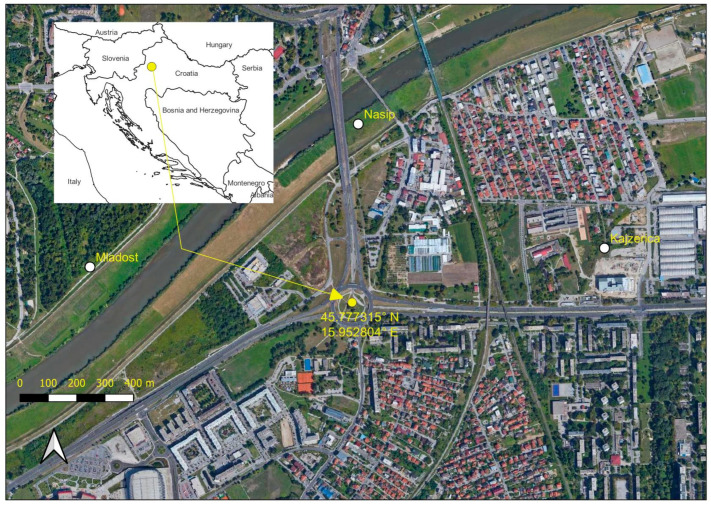
Locations (Mladost, Nasip, Kajzerica) of soil sampling sites for testing the potential impact of Bohemian knotweed on the soil chemistry and allelopathic effects on other plant species. Inner map presents position of Zagreb, Croatia.

The sampling (Figure 4) was based in the Southwest part of the Zagreb, capital of Croatia. The sites are located in the temperate zone (mean annual temperature of 10.8 °C, 880 mm of annual precipitation) at the alluvial floodplains of the Sava River in the area which sustains significant anthropogenic impacts, with mostly human-altered habitats. Sampling was carried out in 2018, at three locations with well-established dense stands of Bohemian knotweed, which were present, based on the historical satellite images, at least from 2008. Knotweed-covered area was around 1500 m^2^ on the Mladost and Nasip sites, and 200 m^2^ on the Kajzerica site (see Figure 4 for the exact position of the localities). 

During our research, we had limited access to the climate chambers in terms of available time and space, and had to adjust our research accordingly. Testing the potential allelopathic effects of Bohemian knotweed was conducted in three stages: 1—germination test in the Petri dishes with knotweed leaf exudates; 2—germination and growth test in the pots with soil with knotweed leaf exudates; 3—germination and growth test in the pots with soil sampled in situ at the knotweed-invaded sites. Healthy and undamaged leaves of Bohemian knotweed for preparing leaf exudates were collected at the Kajzerica locality in the middle of vegetation season (July), while soil samples were collected at the end of the season, in November, at all three localities. In all stages of research, we used as test plants commercially available seeds of mustard (*Sinapis alba* L.) and wheat (*Triticum aestivum* L.). Both species are commonly used as test plants in allelopathy-related research [61,67,68,69]. In all three tests, the seeds were sterilized by soaking in a 1% sodium hypochlorite solution (prepared from the commercial bleach) for 20 min and thoroughly rinsed with distilled water prior to the beginning of the tests.

### 4.1. Germination Test in Petri Dishes with Knotweed Leaf Exudate

The collected leaves of *Reynoutria* × *bohemica* were dried in a Memmert UFB500 universal oven at 70 °C for 48 h. Afterwards, dried leaves were grounded and used for preparation of different concentrations of leaf exudates for allelopathic potential testing. Concentrations were prepared in accordance with the guidelines commonly used in this type of surveys [62,67,70,71,72]. For the initial Petri dish test, we prepared three concentrations (0.01 g/mL; 0.03 g/mL; 0.05 g/mL) using 1, 3 and 5 g of grounded leaf powders that were mixed with 100 mL of distilled water and homogenized with a stick electric mixer. The heterogeneous mixture was transferred into an Erlenmeyer flask and left to soak at room temperature with periodical stirring every 10 min. After an hour, the mixtures were filtered through two layers of sterile gauze into plastic containers with lids. Distilled water was used as negative control. All containers with exudates were kept in the refrigerator.

At this stage, seeds (20 per replicate for each concentration of leaf exudate) of each test plant were transferred into separate plastic containers, into which 10 mL of the exudates was added. Control group of each test plant consisted of additional 20 seeds and 10 mL of distilled water. Everything was prepared and conducted in 3 replications, so a total of 240 seeds of each test plant was used. Seeds were left to soak for 24 h in the dark at room temperature and afterwards were transferred into Petri dishes with Whatman filter paper No. 1, 9 cm in diameter. Into each Petri dish, 5 mL of the corresponding exudate was pipetted, and all samples were transferred into the dark climate chamber for in vitro germination experiment for 5 days at 19 °C. Afterwards, we counted successfully germinated seeds (when the radicle protruded) per test plant and per treatment, and compared them to the control. 

### 4.2. Germination and Growth Tests in the Pots with Soil with Knotweed Leaf Exudate

Based on the obtained results (Table 1), for the germination and growth test in pots with soil we decided to use 0.05 and 0.1 g/mL concentrations of the leaf exudates, prepared in the same manner as described above for the Petri dish test. Seeds of the test plants (*Sinapis alba* and *Triticum aestivum*) were sterilized in the same manner as before and then placed in plastic flowerpots previously filled with a commercially available soil substrate for planting. In total, 17 seeds per pot were used in three replications (a total of 51 seeds per treatment per test plant), each treated with 50 mL of the formerly prepared exudates (i.e., 0.05 and 0.1 g/mL). Distilled water was used in the control treatment (17 seeds per pot per test plant). Sown and watered pots were transferred into the climate chamber where they were grown for 14 days at 24 °C. Germination and growth progresses of the seedlings were checked daily and the pots watered with distilled water if necessary (approximately every other day). On the seventh day, watering was repeated with 50 mL of the corresponding exudate (freshly prepared the previous day). After 14 days, the seedlings were transferred back to the laboratory for measuring.

In each treatment, the number of viable, properly developed mustard and wheat seedlings was recorded, and then their shoots were measured. The seeds that did not develop shoots after the root broke through were not counted as germinated, as well as the seedlings with developmental irregularities. For all properly developed seedlings, fresh shoots were weighed (mg), scanned on an HP Scanjet G3110 scanner, and dried in a a Memmert UFB500 universal oven at 80 °C for 24 h. After drying, the shoots were weighed (mg) again. The length (cm) of the shoots was subsequently measured from the scans using computer program ImageJ ver. 1.43 s.

### 4.3. Germination and Growth Tests in the Pots with Soil Sampled In Situ at the Knotweed-Invaded Sites

**Figure 5:**
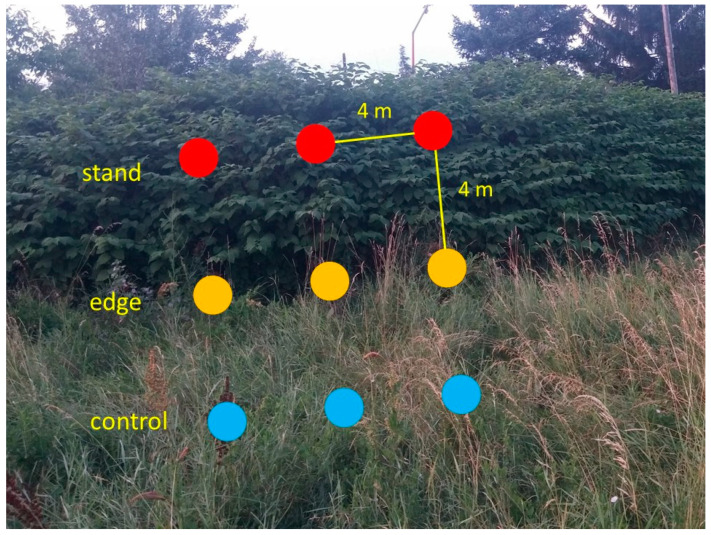
Soil sampling pattern for testing the potential impact of Bohemian knotweed on the soil chemistry and allelopathic effects on other plant species. Photograph taken at Kajzerica site (Photo by Sven D. Jelaska).

To further test the allelopathic potential of Bohemian knotweed, we used soils from three locations with dense stands of *R.* × *bohemica* in Zagreb: at the embankment along the Sava River (Nasip site), at the ruderal habitat in Kajzerica neighborhood and at the Mladost site (Figure 4). In November, at each of these locations, upper 5 cm of the soil was sampled with a cylindrical corer (8 cm in diameter) in a regular 3 × 3 grid (with a distance of 4 m between the neighboring samples) with three samples inside the stand, three at the edge of the stand and three outside the stand as control (Figure 5). Soil samples were on the site directly transferred from the corer into the 250 mL plastic containers used for germination and growth test. Replicates of all samples were collected to determine soil chemical characteristics and transferred to plastic bags, labeled and transported to the laboratory where they were air-dried at room temperature.

Mustard (*S. alba*) and wheat (*T. aestivum*) seeds were used again as test plants for germination and growth experiments, and they were sterilized in the same manner as previously explained. Because of the limitation we had with the available space in the climate chamber, the flowerpots with soil samples were mechanically divided with a plate into two equal sections. Ten mustard and ten wheat seeds (for a total of twenty seeds per pot) were sown in each section, liberally irrigated with distilled water, and then moved to the climate chamber. For the 27 soil samples that were obtained, a total of 540 seeds—270 wheat seeds and 270 mustard seeds—were employed. The seedlings were cultivated for 21 days at 24 °C, with a 2600 lux light intensity and an equal 12 h day and 12 h night cycle of light (light meter PCE-L355). After 21 days (during which the plants were watered with distilled water periodically), the seedlings were transferred back to the laboratory to measure their aboveground fresh mass, shoot length and, after drying in an oven for 24 h at 80 °C, aboveground dry mass. To measure shoot lengths, the samples were scanned and processed with the ImageJ computer program. The latter was completed immediately after weighting and prior to drying. 

We also conducted a germination experiment of the test plants in the same climate chamber and under the same conditions to ascertain the viability of the used seeds. In Petri dishes with a filter paper at the bottom, 20 mustard and 20 wheat seeds were placed in three replications (120 seeds in total) and watered with distilled water. After seven days, the number of germinated test plants was calculated.

### 4.4. Chemical Characteristics of Soil Sampled In Situ at the Knotweed-Invaded Sites

The chemical characteristics of the soil, including pH value, organic matter content, and humus content, were examined using air-dried soil. A combined electrode pH meter (HANNA HI 99121, Direct soil pH meter) was used to measure the pH of suspensions composed of 10 g of soil and 25 mL of distilled water. Prior to measuring, the suspensions were covered for 30 min with occasional stirring. 

Organic matter content was determined by annealing in a muffle furnace (INKO LP-08). According to the NRM Laboratories methodology, Davies 1974, ground soil samples were first dried in an oven at 110 °C for 24 h, then weighed for 5 g samples, transferred to porcelain bowls, and heated in the muffle furnace at 430 °C for 2 h. After annealing, the samples were transferred into a desiccator for cooling and weighted again the next day. The organic matter content was determined using mass differences.

Humus content was determined using the redox titration in potassium permanganate test (Kotzmann method, details in Lab Prototocol1 in the Supplementary Material). 

### 4.5. Statistical Analysis

Statistical analysis was conducted in Statistica 13.5.0.17. (TIBCO Software Inc. (2020). Data Science Workbench, http://tibco.com, accessed on 1 March 2023, 3307 Hillview Avenue Palo Alto, CA 94304, USA). Measured growth parameters of test plants were compared among treatments (i.e., knotweed leaf exudates) and control (i.e., distilled water) and among positions (knotweed stand, edge of stand, control outside of the stand) of sampled soils within three different localities. Soil chemical properties among different positions (knotweed stand, edge of stand, control outside of the stand) of the sampled soil within three different localities were compared. All of the above-mentioned comparisons were conducted with non-parametric Kruskal–Wallis ANOVA and post hoc comparisons of the mean ranks of all pairs of groups, if Kruskal–Wallis test turned out to have a statistically significant result.

## 5. Conclusions

Tests in Petri dishes and pots with soil into which leaf exudates were added showed a decrease in germination and growth in comparison to the control, hence confirming the allelopathic effect. However, this effect was not confirmed in tests with in situ soil samples, where no statistically significant differences were observed in growth of the test plants or chemical characteristics (pH, soil organic matter content, humus content) of the soil. Therefore, the persistence of Bohemian knotweed at the already invaded sites could be attributed to its efficient use of resources (light and nutrients) by which it completely outcompetes native plants. Nevertheless, future research on the allelopathic potential of Bohemian knotweed could compare the presence of this mechanism during different stages of the invasion process (introduction–establishment–dominance), which might enhance our understanding on the topic.

## Figures and Tables

**Table 1:** Results of the germination test of S—white mustard (*Sinapis alba* L.) and T—common wheat (*Triticum aestivum* L.) seeds exposed to the different concentrations of *Reynoutria* × *bohemica* leaf exudates for testing the allelopathic potential. PD—Petri dish test (N = 20 seeds per replicate); Pot—Pot with soil test (N = 17 seeds per replicate); C—control with distilled water.

	Leaf Exudate Concentrations										Control			
Replication	0.01 g/mL (PD)		0.03 g/mL (PD)		0.05 g/mL (PD)		0.05 g/mL (Pot)		0.1 g/mL (Pot)		C (PD)		C (Pot)	
	S	T	S	T	S	T	S	T	S	T	S	T	S	T
1	19	11	20	14	17	4	9	5	7	1	20	15	12	6
2	20	10	19	13	19	8	7	4	0	3	20	17	12	8
3	20	12	18	13	19	7	7	3	7	4	19	17	14	9
% germinated	98.3	55.0	95.0	66.7	91.7	31.7	45.1	24.0	27.5	15.7	98.3	96.1	74.5	45.1
% of the control	100	57.2	96.6	69.4	93.3	33.0	60.5	53.2	36.9	34.8				

**Table 2:** Mean values of measured soil chemical characteristics sampled at three locations with three replicates on each position; inside the stand of *Reynoutria* × *bohemica*, at the edge of the stand and controls outside the stand. *—indicated statistically significant differences (*p* < 0.05) within locality.

Locality	Position	pH	Soil Organic Matter (%)	Humus (%)
Mladost	control	7.69 *	11.33	8.74
Mladost	edge	7.81	7.19	5.33
Mladost	stand	7.90 *	7.50	6.90
Kajzerica	control	7.92	8.79	7.97
Kajzerica	edge	7.75	8.62	7.78
Kajzerica	stand	7.87	8.19	7.69
Nasip	control	7.88	4.92	4.38
Nasip	edge	7.86	4.88	5.26
Nasip	stand	8.00	4.07	4.17

## Data Availability

All data are provided in the Supplementary Materials of this manuscript.

## Supplementary Materials

The following supporting information can be downloaded at: https://www.mdpi.com/article/10.3390/plants12112222/s1, Table S1. Shoot values for 75 germinated 14-day-old *Sinapis alba* seedlings (shoot length, fresh and dry mass and dry/fresh mass ratio) after two treatments (rey5–0.05 g/mL of leaf exudate; rey10–0.1 g/mL of leaf exudate) with *R. × bohemica* leaf exudates and control (distilled water).; Table S2. Shoot values for 75 germinated 14-day-old *Triticum aestivum* seedlings (shoot length, fresh and dry mass and dry/fresh mass ratio) after two treatments (rey5–0.05 g/mL of leaf exudate; rey10–0.1 g/mL of leaf exudate) with *R. × bohemica* leaf exudates and control (distilled water).; Tables S3–S6. Results of the Kruskal–Wallis test and post hoc comparisons of mean ranks of all pairs of groups for comparison of *Sinapis alba* seedlings: shoot length, fresh mass, dry mass and dry/fresh mass ratio after two treatments (rey5–0.05 g/mL of leaf exudate; rey10–0.1 g/mL of leaf exudate) with *R.* × *bohemica* leaf exudates and control (distilled water). All treatments in triplicate with 17 seeds per replicate. Statistically significant values for *p* < 0.05 are printed in red; Tables S7–S10. Results of the Kruskal–Wallis test and post hoc comparisons of mean ranks of all pairs of groups for comparison of *Triticum aestivum* seedlings: shoot length, fresh mass, dry mass and dry/fresh mass ratio after two treatments (rey5–0.05 g/mL of leaf exudate; rey10–0.1 g/mL of leaf exudate) with *R.* × *bohemica* leaf exudates and control (distilled water). All treatments in triplicate with 17 seeds per replicate. Statistically significant values for *p* < 0.05 are printed in red; Table S11. Shoot values for *Sinapis alba* seedlings (shoot length, fresh and dry mass and dry/fresh mass ratio) planted in soil sampled at three locations (Mladost, Kajzerica and Nasip) with three samples on each site, inside the stand of Bohemian knotweed, at the edge of the stand and controls outside the stand.; Table S12. Shoot values for *Triticum aestivum* seedlings (shoot length, fresh and dry mass and dry/fresh mass ratio) planted in soil sampled at three locations (Mladost, Kajzerica and Nasip) with three samples on each site, inside the stand of Bohemian knotweed, at the edge of the stand and controls outside the stand; Table S13. Results of the Kruskal–Wallis test and post hoc comparisons of mean ranks of all pairs of groups for comparison of *Sinapis alba* seedlings. Length grown in pot with soil sampled in triplicates inside the stand, at the edge of the stand and controls outside the stand of *R.* × *bohemica* at locality Mladost. Statistically significant values for *p* < 0.05 are printed in red. Table S14. Results of the Kruskal–Wallis test and post hoc comparisons of mean ranks of all pairs of groups for comparison of *Sinapis alba* seedlings. Dry/fresh mass ratio grown in pot with soil sampled in triplicates inside the stand, at the edge of the stand and controls outside the stand of *R.* × *bohemica* at locality Mladost. Statistically significant values for *p* < 0.05 are printed in red. Table S15. Results of the Kruskal–Wallis test and post hoc comparisons of mean ranks of all pairs of groups for comparison of *Sinapis alba* seedlings. Dry/fresh mass ratio grown in pot with soil sampled in triplicates inside the stand, at the edge of the stand and controls outside the stand of *R.* × *bohemica* at locality Nasip. Statistically significant values for *p* < 0.05 are printed in red. Table S16. Results of the Kruskal–Wallis test and post hoc comparisons of mean ranks of all pairs of groups for comparison of *Triticum aestivum* seedlings. Dry/fresh mass ratio grown in pot with soil sampled in triplicates inside the stand, at the edge of the stand and controls outside the stand of *R.* × *bohemica* at locality Mladost. Statistically significant values for *p* < 0.05 are printed in red. Table S17. Results of the Kruskal–Wallis test and post hoc comparisons of mean ranks of all pairs of groups for comparison of pH value of the soil sampled in triplicates at the location Mladost, inside the stand, at the edge of the stand and controls outside the stand (statistically significant values for *p* < 0.05 are printed in red). Lab protocol LP1. Kotzmann method for humus content determination.
