# Supplementary material for: Bohemian Knotweed Reynoutria × bohemica Chrtek et Chrtková Seems Not to Rely Heavily on Allelopathy for Its Persistence in Invaded Sites in the Southwest Part of the Zagreb, Croatia
Source: Plants (Basel). 2023 Jun 5;12(11):2222. doi: 10.3390/plants12112222 (PMC10255837; doi:10.3390/plants12112222)
Supplement: Supplementary file 1 [file plants-12-02222-s001.zip › plants-2329150-supplementary.pdf]

Supplementary Material

Table S1. Shoot values for 75 germinated 14-day old *Sinapis alba* seedlings (Shoot length, Fresh and Dry mass and Dry/Fresh mass ratio) after two treatments (rey5 – 0.05 g/ml of leaf exudate; rey10 – 0.1 g/ml of leaf exudate) with *R.* × *bohemica* leaf exudates and control (distilled water).

treatment	replicate	length (cm)	mass/fresh (mg)	mass/dry (mg)	dry/fresh*100
rey10	1	5.946	100.7	7.9	7.8
rey10	1	3.29	46.8	5.1	10.9
rey10	1	4.185	87.7	5.7	6.5
rey10	1	3.114	45	3.8	8.4
rey10	1	4.115	52.7	5.2	9.9
rey10	1	2.968	37.6	3.6	9.6
rey10	1	3.775	48.1	6.1	12.7
rey10	3	5.424	73.3	4.7	6.4
rey10	3	3.403	74.6	5	6.7
rey10	3	3.875	51.8	2.8	5.4
rey10	3	4.39	57.9	5.4	9.3
rey10	3	3.882	60.1	4.3	7.2
rey10	3	3.874	59	5.1	8.6
rey10	3	2	19.3	0.5	2.6
rey5	1	6.042	103	5.8	5.6
rey5	1	5.38	97.4	5.4	5.5
rey5	1	4.914	52.9	3.4	6.4
rey5	1	4.499	92.7	6.8	7.3
rey5	1	5.156	87.8	5.1	5.8
rey5	1	3.714	44.7	4.4	9.8
rey5	1	5.898	71.8	4.5	6.3
rey5	1	1.714	35.8	3.3	9.2
rey5	1	2.134	33.5	2.8	8.4
rey5	2	5.498	93.6	6.3	6.7
rey5	2	6.282	94.8	6.1	6.4
rey5	2	3.153	88.3	6.1	6.9
rey5	2	3.052	49.8	3	6.0
rey5	2	3.706	56.1	5.9	10.5
rey5	2	3.577	52	4.5	8.7
rey5	2	4.313	57.6	4.7	8.2
rey5	3	4.873	93.5	6	6.4
rey5	3	4.506	94.8	7.9	8.3
rey5	3	4.357	68.3	5.4	7.9
rey5	3	4.439	89.2	6.8	7.6
rey5	3	4.037	49.2	3.5	7.1
rey5	3	3.82	46	3.6	7.8
rey5	3	3.662	51.3	4.3	8.4
control	1	5.317	123.2	6.4	5.2
control	1	5.786	138.1	6.8	4.9
control	1	6.949	165	8.1	4.9
control	1	5.442	130.2	7.1	5.5

control	1	5.834	119.4	5.8	4.9
control	1	5.01	136.8	6.3	4.6
control	1	5.116	108.5	6.1	5.6
control	1	4.93	70.4	5.1	7.2
control	1	4.062	152.1	7.6	5.0
control	1	4.249	89	4.3	4.8
control	1	3.857	91.3	6.2	6.8
control	1	2.761	56.8	5.3	9.3
control	2	5.026	131.7	7.3	5.5
control	2	4.912	97.1	5.6	5.8
control	2	5.821	127.6	6.5	5.1
control	2	7.374	134.8	7	5.2
control	2	5.641	124.3	6.4	5.1
control	2	4.764	79.5	4.3	5.4
control	2	4.901	85.6	5.2	6.1
control	2	5.582	128.5	6.7	5.2
control	2	3.634	99.4	5.8	5.8
control	2	4.576	78.4	5.9	7.5
control	2	2.967	66.1	4.1	6.2
control	2	2.304	39.5	3.5	8.9
control	3	5.341	172.1	8.2	4.8
control	3	5.791	171.4	8.8	5.1
control	3	4.648	155.8	7.7	4.9
control	3	5.832	144	7.3	5.1
control	3	5.59	115.4	5.7	4.9
control	3	5.285	156.7	8.2	5.2
control	3	4.651	126.1	6.4	5.1
control	3	5.27	160.9	8.6	5.3
control	3	3.882	126.2	7.4	5.9
control	3	4.358	104.3	5.4	5.2
control	3	2.992	76.5	5.6	7.3
control	3	3.04	55.5	3.9	7.0
control	3	2.821	55.5	5.9	10.6
control	3	1.848	38.1	4.9	12.9

Table S2. Shoot values for 75 germinated 14-day old *Triticum aestivum* seedlings (Shoot length, Fresh and Dry mass and Dry/Fresh mass ratio) after two treatments (rey5 – 0.05 g/ml of leaf exudate; rey10 – 0.1 g/ml of leaf exudate) with *R.* × *bohemica* leaf exudates and control (distilled water).

treatment	replicate	length (cm)	mass/fresh (mg)	mass/dry (mg)	dry/fresh*100
rey10	1	15.944	93.2	10.6	11.4
rey10	2	14.138	73.9	8.3	11.2
rey10	2	19.268	127.9	14.2	11.1
rey10	2	15.277	95	11.3	11.9
rey10	3	16.005	129	16.3	12.6
rey10	3	15.084	93.2	11.4	12.2
rey10	3	12.291	56.3	6.8	12.1
rey10	3	9.714	62.2	7.8	12.5
rey5	1	20.555	175.3	21.1	12.0
rey5	1	18.454	161	18.9	11.7
rey5	1	8.673	48	5.9	12.3
rey5	1	6.231	45.9	5	10.9
rey5	1	3.211	17	1.6	9.4
rey5	2	18.829	153.2	17.3	11.3
rey5	2	11.809	65.6	7.1	10.8
rey5	2	11.009	77.5	8.7	11.2
rey5	2	2.6	19.3	2.4	12.4
rey5	3	17.295	151	18.8	12.5
rey5	3	11.79	75.2	8.9	11.8
rey5	3	9.29	54.6	6.3	11.5
control	1	24.861	221.2	21.9	9.9
control	1	22.893	259	24.2	9.3
control	1	19.799	176.7	18	10.2
control	1	15.804	131.7	15.8	12.0
control	1	16.191	119.7	11.4	9.5
control	1	11.439	87.8	10	11.4
control	2	28.123	257.5	23.9	9.3
control	2	21.582	210	21	10.0
control	2	19.099	171.6	17	9.9
control	2	20.842	206.6	19.5	9.4
control	2	16.432	152.8	17.6	11.5
control	2	15.457	160.9	15.8	9.8
control	2	16.654	156.7	15.7	10.0
control	2	10.707	75.4	8.2	10.9
control	3	22.24	179.2	18.9	10.5
control	3	17.051	140.1	13.6	9.7
control	3	18.344	169.1	19.2	11.4
control	3	15.786	143	15.5	10.8
control	3	16.06	122.9	11.9	9.7
control	3	13.517	125.4	13.4	10.7
control	3	12.247	53.4	5.8	10.9
control	3	12.657	93.3	9.5	10.2
control	3	8.94	49.8	5	10.0

Table S3. Results of the Kruskal-Wallis test and post-hoc comparisons of mean ranks of all pairs of groups for comparison of *Sinapis alba* seedlings Shoot length after two treatments (rey5 – 0.05 g/ml of leaf exudate; rey10 – 0.1 g/ml of leaf exudate) with *R.* × *bohemica* leaf exudates and control (distilled water). All treatments in triplicate with 17 seeds per replicate. Statistically significant values for p <0.05 are printed in red.

Depend.: length (cm)	Kruskal-Wallis test: H (2, N= 75) =5.468829 p =.0649			
	Code	Valid N	Sum of Ranks	Mean Rank
rey10	1	14	386.500	27.60714
rey5	2	23	826.000	35.91304
control	3	38	1637.500	43.09211

Depend.: length (cm)	Multiple Comparisons z' values - upper right part<br>Multiple Comparisons p values (2-tailed) - lower left part		
	rey10	rey5	control
rey10		1.124261	2.272571
rey5	0.782707		1.246843
control	0.069156	0.637365	

Table S4. Results of the Kruskal-Wallis test and post-hoc comparisons of mean ranks of all pairs of groups for comparison of *Sinapis alba* seedlings Fresh mass after two treatments (rey5 – 0.05 g/ml of leaf exudate; rey10 – 0.1 g/ml of leaf exudate) with *R.* × *bohemica* leaf exudates and control (distilled water). All treatments in triplicate with 17 seeds per replicate. Statistically significant values for p <0.05 are printed in red.

Depend.: mass/fresh (mg)	Kruskal-Wallis test: H (2, N= 75) =26.12181 p =0.0000			
	Code	Valid N	Sum of Ranks	Mean Rank
rey10	1	14	292.000	20.85714
rey5	2	23	640.000	27.82609
control	3	38	1918.000	50.47368

Depend.: mass/fresh (mg)	Multiple Comparisons z' values - upper right part<br>Multiple Comparisons p values (2-tailed) - lower left part		
	rey10	rey5	control
rey10		0.943294	4.346520
rey5	1.000000		3.933382
control	0.000041	0.000251	

Table S5. Results of the Kruskal-Wallis test and post-hoc comparisons of mean ranks of all pairs of groups for comparison of *Sinapis alba* seedlings Dry mass after two treatments (rey5 – 0.05 g/ml of leaf exudate; rey10 – 0.1 g/ml of leaf exudate) with *R.* × *bohemica* leaf exudates and control (distilled water). All treatments in triplicate with 17 seeds per replicate. Statistically significant values for p <0.05 are printed in red.

Depend.: mass/dry (mg)	Kruskal-Wallis test: H (2, N= 75) =14.66624 p =0.0007			
	Code	Valid N	Sum of Ranks	Mean Rank
rey10	1	14	357.500	25.53571
rey5	2	23	692.000	30.08696
control	3	38	1800.500	47.38158

Depend.: mass/dry (mg)	Multiple Comparisons z' values - upper right part<br>Multiple Comparisons p values (2-tailed) - lower left part		
	rey10	rey5	control
rey10		0.616042	3.206096
rey5	1.000000		3.003690
control	0.004036	0.008002	

Table S6. Results of the Kruskal-Wallis test and post-hoc comparisons of mean ranks of all pairs of groups for comparison of *Sinapis alba* seedlings Dry/Fresh mass ratio after two treatments (rey5 – 0.05 g/ml of leaf exudate; rey10 – 0.1 g/ml of leaf exudate) with *R.* × *bohemica* leaf exudates and control (distilled water). All treatments in triplicate with 17 seeds per replicate. Statistically significant values for p <0.05 are printed in red.

Depend.: dry/fresh*100	Kruskal-Wallis test: H (2, N= 75) =21.38261 p =0.0000			
	Code	Valid N	Sum of Ranks	Mean Rank
rey10	1	14	720.000	51.42857
rey5	2	23	1121.000	48.73913
control	3	38	1009.000	26.55263

Depend.: dry/fresh*100	Multiple Comparisons z' values - upper right part<br>Multiple Comparisons p values (2-tailed) - lower left part		
	rey10	rey5	control
rey10		0.364034	3.650790
rey5	1.000000		3.853300
control	0.000784	0.000350	

Table S7. Results of the Kruskal-Wallis test and post-hoc comparisons of mean ranks of all pairs of groups for comparison of *Triticum aestivum* seedlings Shoot length after two treatments (rey5 – 0.05 g/ml of leaf exudate; rey10 – 0.1 g/ml of leaf exudate) with *R.* × *bohemica* leaf exudates and control (distilled water). All treatments in triplicate with 17 seeds per replicate. Statistically significant values for p <0.05 are printed in red.

Depend.: length (cm)	Kruskal-Wallis test: H (2, N= 43) =6.941137 p =0.0311			
	Code	Valid N	Sum of Ranks	Mean Rank
rey10	1	8	157.000	19.62500
rey5	2	12	180.000	15.00000
control	3	23	609.000	26.47826

Depend.: length (cm)	Multiple Comparisons z' values - upper right part<br>Multiple Comparisons p values (2-tailed) - lower left part		
	rey10	rey5	control
rey10		0.806979	1.329705
rey5	1.000000		2.567005
control	0.550846	0.030774	

Table S8. Results of the Kruskal-Wallis test and post-hoc comparisons of mean ranks of all pairs of groups for comparison of *Triticum aestivum* seedlings Fresh mass after two treatments (rey5 – 0.05 g/ml of leaf exudate; rey10 – 0.1 g/ml of leaf exudate) with *R.* × *bohemica* leaf exudates and control (distilled water). All treatments in triplicate with 17 seeds per replicate. Statistically significant values for p <0.05 are printed in red.

Depend.: mass/fresh (mg)	Kruskal-Wallis test: H (2, N= 43) =10.51165 p =0.0052			
	Code	Valid N	Sum of Ranks	Mean Rank
rey10	1	8	127.000	15.87500
rey5	2	12	180.000	15.00000
control	3	23	639.000	27.78261

Depend.: mass/fresh (mg)	Multiple Comparisons z' values - upper right part<br>Multiple Comparisons p values (2-tailed) - lower left part		
	rey10	rey5	control
rey10		0.152672	2.310376
rey5	1.000000		2.858710
control	0.062602	0.012761	

Table S9. Results of the Kruskal-Wallis test and post-hoc comparisons of mean ranks of all pairs of groups for comparison of *Triticum aestivum* seedlings Dry mass after two treatments (rey5 – 0.05 g/ml of leaf exudate; rey10 – 0.1 g/ml of leaf exudate) with *R.* × *bohemica* leaf exudates and control (distilled water). All treatments in triplicate with 17 seeds per replicate. Statistically significant values for p <0.05 are printed in red.

Depend.: mass/dry (mg)	Kruskal-Wallis test: H (2, N= 43) =6.748904 p =0.0342			
	Code	Valid N	Sum of Ranks	Mean Rank
rey10	1	8	137.500	17.18750
rey5	2	12	196.000	16.33333
control	3	23	612.500	26.63043

Depend.: mass/dry (mg)	Multiple Comparisons z' values - upper right part<br>Multiple Comparisons p values (2-tailed) - lower left part		
	rey10	rey5	control
rey10		0.149037	1.832167
rey5	1.000000		2.302850
control	0.200780	0.063862	

Table S10. Results of the Kruskal-Wallis test and post-hoc comparisons of mean ranks of all pairs of groups for comparison of *Triticum aestivum* seedlings Dry/Fresh mass ratio after two treatments (rey5 – 0.05 g/ml of leaf exudate; rey10 – 0.1 g/ml of leaf exudate) with *R.* × *bohemica* leaf exudates and control (distilled water). All treatments in triplicate with 17 seeds per replicate. Statistically significant values for p <0.05 are printed in red.

Depend.: dry/fresh*100	Kruskal-Wallis test: H (2, N= 43) =18.91821 p =0.0001			
	Code	Valid N	Sum of Ranks	Mean Rank
rey10	1	8	270.000	33.75000
rey5	2	12	345.000	28.75000
control	3	23	331.000	14.39130

Depend.: dry/fresh*100	Multiple Comparisons z' values - upper right part<br>Multiple Comparisons p values (2-tailed) - lower left part		
	rey10	rey5	control
rey10		0.872410	3.756075
rey5	1.000000		3.211187
control	0.000518	0.003966	

Table S11. Shoot values for *Sinapis alba* seedlings (Shoot length, Fresh and Dry mass and Dry/Fresh mass ratio) planted in soil sampled at three locations (Mladost, Kajzerica and Nasip) with three samples on each site, inside the stand of the Bohemian knotweed, at the edge of the stand and controls outside the stand.

locality	position	length (cm)	mass fresh [g]	mass dry [g]	mass ratio dry/fresh
mladost	stand	1.49	0.026	0.006	0.2308
mladost	stand	1.89	0.023	0.004	0.1739
mladost	stand	6.30	0.146	0.010	0.0685
mladost	stand	7.81	0.127	0.007	0.0551
mladost	stand	7.77	0.115	0.005	0.0435
mladost	stand	8.98	0.212	0.012	0.0566
mladost	stand	9.61	0.230	0.012	0.0522
mladost	stand	4.54	0.066	0.002	0.0303
mladost	stand	8.00	0.114	0.006	0.0526
mladost	stand	6.60	0.124	0.008	0.0645
mladost	stand	8.62	0.146	0.009	0.0616
mladost	stand	8.37	0.131	0.008	0.0611
mladost	stand	2.27	0.019	0.004	0.2105
mladost	stand	3.06	0.054	0.006	0.1111
mladost	stand	9.01	0.144	0.010	0.0694
mladost	stand	7.01	0.126	0.008	0.0635
mladost	stand	8.38	0.140	0.006	0.0429
mladost	stand	8.22	0.094	0.005	0.0532
mladost	stand	8.81	0.116	0.008	0.0690
mladost	stand	7.80	0.117	0.008	0.0684
mladost	stand	6.53	0.126	0.007	0.0556
mladost	stand	5.95	0.114	0.006	0.0526
mladost	edge	7.99	0.142	0.010	0.0704
mladost	edge	9.25	0.127	0.008	0.0630
mladost	edge	7.63	0.105	0.004	0.0381
mladost	edge	8.12	0.095	0.005	0.0526
mladost	edge	9.19	0.151	0.011	0.0728
mladost	edge	8.19	0.149	0.010	0.0671
mladost	edge	7.74	0.121	0.008	0.0661
mladost	edge	8.35	0.147	0.010	0.0680
mladost	edge	6.51	0.097	0.006	0.0619
mladost	edge	8.38	0.164	0.011	0.0671
mladost	edge	10.38	0.140	0.007	0.0500
mladost	edge	7.93	0.122	0.005	0.0410
mladost	edge	8.47	0.154	0.010	0.0649
mladost	edge	9.62	0.163	0.010	0.0613
mladost	edge	8.80	0.124	0.005	0.0403
mladost	edge	9.25	0.110	0.005	0.0455
mladost	edge	10.46	0.160	0.010	0.0625
mladost	edge	8.39	0.139	0.007	0.0504
mladost	edge	8.24	0.128	0.006	0.0469
mladost	edge	9.97	0.148	0.007	0.0473
mladost	edge	8.52	0.181	0.009	0.0497
mladost	edge	9.01	0.183	0.010	0.0546

mladost	edge	9.87	0.161	0.007	0.0435
mladost	edge	8.01	0.100	0.005	0.0500
mladost	control	6.76	0.130	0.007	0.0538
mladost	control	8.90	0.161	0.009	0.0559
mladost	control	8.71	0.132	0.008	0.0606
mladost	control	7.05	0.185	0.012	0.0649
mladost	control	4.67	0.057	0.004	0.0702
mladost	control	8.56	0.119	0.006	0.0504
mladost	control	7.53	0.104	0.005	0.0481
mladost	control	4.93	0.150	0.008	0.0533
mladost	control	8.58	0.126	0.010	0.0794
mladost	control	8.10	0.136	0.010	0.0735
mladost	control	6.05	0.178	0.013	0.0730
mladost	control	8.21	0.069	0.004	0.0580
mladost	control	5.89	0.086	0.006	0.0698
mladost	control	6.37	0.110	0.008	0.0727
mladost	control	8.26	0.112	0.008	0.0714
mladost	control	6.83	0.115	0.009	0.0783
kajzerica	control	1.65	0.025	0.003	0.1200
kajzerica	control	1.83	0.025	0.003	0.1200
kajzerica	control	1.56	0.025	0.002	0.0800
kajzerica	control	4.06	0.078	0.008	0.1026
kajzerica	control	9.85	0.377	0.017	0.0451
kajzerica	control	8.82	0.161	0.010	0.0621
kajzerica	control	8.08	0.172	0.011	0.0640
kajzerica	control	6.85	0.133	0.008	0.0602
kajzerica	control	8.94	0.210	0.014	0.0667
kajzerica	control	6.35	0.206	0.012	0.0583
kajzerica	control	7.93	0.190	0.012	0.0632
kajzerica	control	7.63	0.164	0.010	0.0610
kajzerica	control	7.63	0.106	0.006	0.0566
kajzerica	control	8.75	0.206	0.011	0.0534
kajzerica	control	4.03	0.062	0.003	0.0484
kajzerica	control	4.62	0.082	0.004	0.0488
kajzerica	control	7.63	0.216	0.010	0.0463
kajzerica	control	8.62	0.229	0.012	0.0524
kajzerica	control	10.00	0.256	0.012	0.0469
kajzerica	control	8.36	0.258	0.013	0.0504
kajzerica	control	8.74	0.174	0.007	0.0402
kajzerica	stand	6.53	0.132	0.008	0.0606
kajzerica	stand	7.47	0.139	0.009	0.0647
kajzerica	stand	8.08	0.214	0.014	0.0654
kajzerica	stand	7.90	0.131	0.008	0.0611
kajzerica	stand	8.66	0.132	0.009	0.0682
kajzerica	stand	8.61	0.116	0.006	0.0517
kajzerica	stand	7.32	0.098	0.005	0.0510
kajzerica	stand	4.99	0.070	0.004	0.0571
kajzerica	stand	5.19	0.068	0.005	0.0735

kajzerica	stand	3.71	0.051	0.003	0.0588
kajzerica	stand	7.98	0.151	0.007	0.0464
kajzerica	stand	10.25	0.138	0.012	0.0870
kajzerica	stand	7.58	0.135	0.005	0.0370
kajzerica	stand	7.19	0.158	0.007	0.0443
kajzerica	stand	10.16	0.159	0.008	0.0503
kajzerica	stand	7.25	0.092	0.005	0.0543
kajzerica	stand	9.90	0.140	0.008	0.0571
kajzerica	stand	7.48	0.111	0.006	0.0541
kajzerica	stand	8.77	0.142	0.007	0.0493
kajzerica	stand	7.56	0.101	0.006	0.0594
kajzerica	stand	6.91	0.251	0.010	0.0398
kajzerica	stand	8.12	0.153	0.008	0.0523
kajzerica	stand	11.25	0.131	0.014	0.1069
kajzerica	stand	8.26	0.247	0.016	0.0648
kajzerica	stand	10.82	0.173	0.012	0.0694
kajzerica	stand	8.19	0.142	0.007	0.0493
kajzerica	stand	7.86	0.140	0.006	0.0429
kajzerica	stand	7.13	0.152	0.008	0.0526
kajzerica	stand	8.08	0.162	0.009	0.0556
kajzerica	edge	8.19	0.172	0.011	0.0640
kajzerica	edge	6.81	0.132	0.006	0.0455
kajzerica	edge	7.35	0.132	0.006	0.0455
kajzerica	edge	7.12	0.149	0.008	0.0537
kajzerica	edge	7.88	0.131	0.007	0.0534
kajzerica	edge	7.82	0.126	0.007	0.0556
kajzerica	edge	5.50	0.134	0.006	0.0448
kajzerica	edge	5.55	0.107	0.004	0.0374
kajzerica	edge	6.80	0.131	0.006	0.0458
kajzerica	edge	5.91	0.117	0.005	0.0427
kajzerica	edge	9.89	0.238	0.013	0.0546
kajzerica	edge	8.84	0.188	0.012	0.0638
kajzerica	edge	7.87	0.164	0.012	0.0732
kajzerica	edge	7.81	0.192	0.014	0.0729
kajzerica	edge	7.70	0.167	0.008	0.0479
kajzerica	edge	9.08	0.253	0.018	0.0711
kajzerica	edge	8.70	0.202	0.011	0.0545
kajzerica	edge	7.45	0.157	0.007	0.0446
kajzerica	edge	7.83	0.148	0.011	0.0743
kajzerica	edge	7.34	0.119	0.006	0.0504
kajzerica	edge	7.70	0.130	0.006	0.0462
kajzerica	edge	9.37	0.259	0.020	0.0772
kajzerica	edge	8.75	0.143	0.007	0.0490
kajzerica	edge	6.14	0.129	0.009	0.0698
kajzerica	edge	9.46	0.147	0.009	0.0612
kajzerica	edge	5.32	0.199	0.011	0.0553
kajzerica	edge	9.34	0.146	0.007	0.0479
kajzerica	edge	7.05	0.136	0.005	0.0368

kajzerica	edge	5.77	0.094	0.003	0.0319
nasip	control	7.69	0.162	0.008	0.0494
nasip	control	8.00	0.149	0.008	0.0537
nasip	control	7.28	0.156	0.009	0.0577
nasip	control	6.31	0.155	0.009	0.0581
nasip	control	7.31	0.111	0.005	0.0450
nasip	control	10.16	0.209	0.014	0.0670
nasip	control	4.42	0.057	0.004	0.0702
nasip	control	8.00	0.183	0.012	0.0656
nasip	control	7.80	0.164	0.011	0.0671
nasip	control	7.65	0.160	0.010	0.0625
nasip	control	8.37	0.141	0.008	0.0567
nasip	control	9.59	0.139	0.007	0.0504
nasip	control	9.65	0.138	0.007	0.0507
nasip	control	6.91	0.169	0.011	0.0651
nasip	control	3.03	0.023	0.003	0.1304
nasip	edge	6.96	0.139	0.011	0.0791
nasip	edge	6.84	0.155	0.012	0.0774
nasip	edge	5.98	0.099	0.005	0.0505
nasip	edge	7.32	0.131	0.010	0.0763
nasip	edge	7.28	0.141	0.011	0.0780
nasip	edge	8.41	0.151	0.012	0.0795
nasip	edge	8.04	0.162	0.013	0.0802
nasip	edge	6.71	0.115	0.008	0.0696
nasip	edge	7.40	0.153	0.011	0.0719
nasip	edge	7.04	0.129	0.008	0.0620
nasip	edge	7.21	0.116	0.007	0.0603
nasip	edge	6.64	0.100	0.007	0.0700
nasip	edge	8.10	0.156	0.011	0.0705
nasip	edge	7.30	0.135	0.009	0.0667
nasip	edge	6.41	0.092	0.004	0.0435
nasip	edge	6.68	0.129	0.009	0.0698
nasip	edge	8.00	0.149	0.011	0.0738
nasip	edge	5.99	0.148	0.010	0.0676
nasip	edge	8.18	0.183	0.014	0.0765
nasip	edge	7.58	0.145	0.011	0.0759
nasip	edge	6.81	0.075	0.005	0.0667
nasip	edge	6.93	0.170	0.012	0.0706
nasip	edge	7.09	0.139	0.009	0.0647
nasip	edge	8.95	0.166	0.012	0.0723
nasip	edge	6.43	0.129	0.008	0.0620
nasip	stand	8.66	0.135	0.008	0.0593
nasip	stand	8.18	0.130	0.008	0.0615
nasip	stand	8.42	0.144	0.012	0.0833
nasip	stand	8.00	0.121	0.007	0.0579
nasip	stand	7.03	0.122	0.007	0.0574
nasip	stand	6.72	0.139	0.011	0.0791
nasip	stand	5.35	0.143	0.012	0.0839

nasip	stand	4.21	0.067	0.006	0.0896
nasip	stand	5.78	0.121	0.008	0.0661
nasip	stand	7.62	0.201	0.014	0.0697
nasip	stand	7.79	0.196	0.014	0.0714
nasip	stand	6.79	0.209	0.015	0.0718
nasip	stand	6.01	0.142	0.010	0.0704
nasip	stand	4.03	0.057	0.003	0.0526
nasip	stand	1.05	0.013	0.001	0.0769
nasip	stand	8.24	0.174	0.013	0.0747
nasip	stand	8.44	0.157	0.010	0.0637
nasip	stand	7.40	0.182	0.014	0.0769

Table S12. Shoot values for *Triticum aestivum* seedlings (Shoot length, Fresh and Dry mass and Dry/Fresh mass ratio) planted in soil sampled at three locations (Mladost, Kajzerica and Nasip) with three samples on each site, inside the stand of the Bohemian knotweed, at the edge of the stand and controls outside the stand.

locality	position	length (cm)	mass fresh [g]	mass dry [g]	mass ratio dry/fresh
mladost	stand	20.84	0.120	0.013	0.1083
mladost	stand	20.49	0.136	0.016	0.1176
mladost	stand	32.73	0.246	0.030	0.1220
mladost	stand	2.39	0.020	0.002	0.1000
mladost	stand	21.91	0.205	0.023	0.1122
mladost	stand	24.45	0.185	0.020	0.1081
mladost	stand	20.97	0.142	0.016	0.1127
mladost	stand	17.75	0.124	0.013	0.1048
mladost	stand	17.48	0.096	0.004	0.0417
mladost	stand	21.20	0.168	0.017	0.1012
mladost	stand	23.19	0.163	0.017	0.1043
mladost	stand	21.97	0.142	0.016	0.1127
mladost	stand	21.58	0.160	0.019	0.1188
mladost	stand	21.59	0.182	0.023	0.1264
mladost	stand	17.47	0.113	0.012	0.1062
mladost	stand	20.00	0.181	0.024	0.1326
mladost	stand	18.38	0.119	0.014	0.1176
mladost	stand	18.58	0.139	0.015	0.1079
mladost	stand	20.08	0.138	0.015	0.1087
mladost	stand	18.01	0.129	0.014	0.1085
mladost	stand	22.01	0.155	0.020	0.1290
mladost	stand	19.22	0.131	0.015	0.1145
mladost	stand	22.75	0.180	0.022	0.1222
mladost	edge	19.19	0.155	0.020	0.1290
mladost	edge	17.82	0.125	0.017	0.1360
mladost	edge	23.58	0.193	0.023	0.1192
mladost	edge	22.92	0.150	0.020	0.1333
mladost	edge	21.64	0.155	0.021	0.1355
mladost	edge	19.31	0.156	0.020	0.1282
mladost	edge	22.56	0.143	0.017	0.1189
mladost	edge	17.85	0.133	0.015	0.1128
mladost	edge	22.78	0.146	0.019	0.1301
mladost	edge	23.44	0.181	0.022	0.1215
mladost	edge	17.26	0.117	0.015	0.1282
mladost	edge	18.66	0.134	0.018	0.1343
mladost	edge	22.12	0.178	0.020	0.1124
mladost	edge	19.66	0.136	0.017	0.1250
mladost	edge	20.65	0.166	0.018	0.1084
mladost	edge	18.25	0.130	0.011	0.0846
mladost	edge	20.08	0.133	0.014	0.1053
mladost	edge	23.46	0.213	0.022	0.1033
mladost	edge	17.53	0.146	0.020	0.1370
mladost	edge	26.38	0.231	0.026	0.1126
mladost	edge	17.08	0.110	0.014	0.1273

mladost	edge	23.28	0.177	0.022	0.1243
mladost	edge	20.94	0.105	0.015	0.1429
mladost	edge	22.22	0.165	0.018	0.1091
mladost	control	20.05	0.141	0.016	0.1135
mladost	control	28.40	0.256	0.026	0.1016
mladost	control	24.10	0.182	0.023	0.1264
mladost	control	28.19	0.266	0.032	0.1203
mladost	control	1.39	0.010	0.001	0.1000
mladost	control	25.70	0.183	0.022	0.1202
mladost	control	25.79	0.157	0.019	0.1210
mladost	control	12.84	0.093	0.014	0.1505
mladost	control	2.82	0.022	0.004	0.1818
mladost	control	11.60	0.085	0.013	0.1529
mladost	control	13.99	0.102	0.016	0.1569
mladost	control	1.74	0.016	0.003	0.1875
mladost	control	25.65	0.163	0.023	0.1411
mladost	control	21.04	0.128	0.018	0.1406
mladost	control	25.78	0.189	0.022	0.1164
mladost	control	16.74	0.096	0.015	0.1563
mladost	control	19.49	0.128	0.020	0.1563
mladost	control	24.81	0.206	0.029	0.1408
mladost	control	16.57	0.119	0.017	0.1429
mladost	control	19.73	0.150	0.019	0.1267
kajzerica	control	1.59	0.015	0.003	0.2000
kajzerica	control	2.68	0.025	0.005	0.2000
kajzerica	control	27.26	0.263	0.027	0.1027
kajzerica	control	26.85	0.297	0.029	0.0976
kajzerica	control	24.14	0.221	0.025	0.1131
kajzerica	control	25.79	0.255	0.027	0.1059
kajzerica	control	26.62	0.210	0.024	0.1143
kajzerica	control	25.59	0.226	0.025	0.1106
kajzerica	control	21.75	0.153	0.018	0.1176
kajzerica	control	27.25	0.195	0.022	0.1128
kajzerica	control	23.80	0.185	0.020	0.1081
kajzerica	control	24.06	0.189	0.020	0.1058
kajzerica	control	20.86	0.146	0.010	0.0685
kajzerica	control	23.24	0.186	0.023	0.1237
kajzerica	control	18.83	0.123	0.015	0.1220
kajzerica	control	17.60	0.114	0.009	0.0789
kajzerica	control	6.69	0.039	0.005	0.1282
kajzerica	control	24.83	0.200	0.026	0.1300
kajzerica	control	21.46	0.150	0.015	0.1000
kajzerica	control	24.54	0.165	0.017	0.1030
kajzerica	control	23.62	0.166	0.017	0.1024
kajzerica	control	31.60	0.215	0.027	0.1256
kajzerica	control	16.21	0.099	0.012	0.1212
kajzerica	control	23.34	0.187	0.018	0.0963
kajzerica	control	23.20	0.186	0.018	0.0968

kajzerica	control	16.34	0.068	0.005	0.0735
kajzerica	stand	26.26	0.198	0.023	0.1162
kajzerica	stand	25.56	0.226	0.025	0.1106
kajzerica	stand	25.34	0.224	0.023	0.1027
kajzerica	stand	23.49	0.165	0.018	0.1091
kajzerica	stand	27.19	0.273	0.027	0.0989
kajzerica	stand	21.43	0.179	0.020	0.1117
kajzerica	stand	20.99	0.138	0.016	0.1159
kajzerica	stand	19.94	0.144	0.017	0.1181
kajzerica	stand	14.20	0.056	0.006	0.1071
kajzerica	stand	10.19	0.075	0.009	0.1200
kajzerica	stand	20.06	0.137	0.017	0.1241
kajzerica	stand	20.66	0.142	0.013	0.0915
kajzerica	stand	25.54	0.217	0.025	0.1152
kajzerica	stand	20.90	0.146	0.014	0.0959
kajzerica	stand	24.77	0.165	0.019	0.1152
kajzerica	stand	21.16	0.150	0.017	0.1133
kajzerica	stand	22.80	0.182	0.020	0.1099
kajzerica	stand	23.25	0.163	0.019	0.1166
kajzerica	stand	24.31	0.195	0.021	0.1077
kajzerica	stand	21.33	0.162	0.015	0.0926
kajzerica	stand	24.93	0.223	0.023	0.1031
kajzerica	stand	26.38	0.188	0.018	0.0957
kajzerica	stand	26.26	0.229	0.023	0.1004
kajzerica	stand	21.66	0.162	0.015	0.0926
kajzerica	stand	20.85	0.168	0.016	0.0952
kajzerica	stand	25.40	0.222	0.021	0.0946
kajzerica	stand	0.00	0.006	0.001	0.1667
kajzerica	edge	21.52	0.164	0.018	0.1098
kajzerica	edge	22.38	0.170	0.019	0.1118
kajzerica	edge	20.87	0.160	0.016	0.1000
kajzerica	edge	25.34	0.227	0.027	0.1189
kajzerica	edge	24.10	0.209	0.022	0.1053
kajzerica	edge	23.00	0.192	0.020	0.1042
kajzerica	edge	15.42	0.121	0.012	0.0992
kajzerica	edge	18.09	0.143	0.017	0.1189
kajzerica	edge	25.21	0.256	0.028	0.1094
kajzerica	edge	26.39	0.308	0.032	0.1039
kajzerica	edge	27.45	0.270	0.029	0.1074
kajzerica	edge	25.11	0.253	0.024	0.0949
kajzerica	edge	24.51	0.169	0.013	0.0769
kajzerica	edge	24.66	0.175	0.014	0.0800
kajzerica	edge	25.23	0.252	0.024	0.0952
kajzerica	edge	26.51	0.204	0.020	0.0980
kajzerica	edge	25.00	0.288	0.033	0.1146
kajzerica	edge	25.00	0.183	0.016	0.0874
kajzerica	edge	25.98	0.213	0.023	0.1080
kajzerica	edge	24.34	0.188	0.019	0.1011

kajzerica	edge	25.76	0.214	0.022	0.1028
kajzerica	edge	21.94	0.163	0.016	0.0982
kajzerica	edge	24.89	0.237	0.026	0.1097
kajzerica	edge	26.63	0.215	0.023	0.1070
kajzerica	edge	14.68	0.096	0.012	0.1250
nasip	control	22.73	0.167	0.019	0.1138
nasip	control	19.39	0.121	0.013	0.1074
nasip	control	11.73	0.075	0.010	0.1333
nasip	control	9.88	0.065	0.008	0.1231
nasip	control	12.74	0.104	0.012	0.1154
nasip	control	20.49	0.196	0.022	0.1122
nasip	control	26.73	0.193	0.018	0.0933
nasip	control	27.17	0.232	0.022	0.0948
nasip	control	14.44	0.126	0.014	0.1111
nasip	control	15.17	0.111	0.011	0.0991
nasip	control	10.19	0.052	0.006	0.1154
nasip	edge	24.91	0.185	0.024	0.1297
nasip	edge	20.85	0.182	0.024	0.1319
nasip	edge	20.67	0.125	0.018	0.1440
nasip	edge	18.55	0.144	0.020	0.1389
nasip	edge	16.58	0.128	0.017	0.1328
nasip	edge	17.27	0.128	0.016	0.1250
nasip	edge	18.14	0.148	0.016	0.1081
nasip	edge	21.50	0.155	0.018	0.1161
nasip	edge	19.74	0.145	0.016	0.1103
nasip	edge	21.57	0.184	0.020	0.1087
nasip	edge	19.93	0.142	0.020	0.1408
nasip	edge	21.78	0.196	0.022	0.1122
nasip	edge	18.79	0.128	0.018	0.1406
nasip	edge	18.21	0.091	0.015	0.1648
nasip	edge	24.12	0.174	0.018	0.1034
nasip	edge	19.99	0.124	0.013	0.1048
nasip	edge	21.56	0.154	0.015	0.0974
nasip	edge	20.21	0.137	0.014	0.1022
nasip	edge	15.19	0.091	0.011	0.1209
nasip	edge	2.85	0.021	0.003	0.1429
nasip	stand	23.85	0.205	0.025	0.1220
nasip	stand	24.36	0.202	0.022	0.1089
nasip	stand	19.72	0.142	0.015	0.1056
nasip	stand	22.88	0.189	0.020	0.1058
nasip	stand	19.03	0.124	0.013	0.1048
nasip	stand	19.04	0.139	0.015	0.1079
nasip	stand	20.52	0.174	0.016	0.0920
nasip	stand	16.42	0.105	0.012	0.1143
nasip	stand	9.69	0.058	0.008	0.1379
nasip	stand	5.33	0.035	0.004	0.1143
nasip	stand	24.21	0.205	0.022	0.1073
nasip	stand	21.54	0.146	0.017	0.1164

nasip	stand	20.85	0.211	0.023	0.1090
nasip	stand	21.42	0.210	0.025	0.1190

Table S13. Results of the Kruskal-Wallis test and post-hoc comparisons of mean ranks of all pairs of groups for comparison of *Sinapis alba* seedlings Length grown in pot with soil sampled in triplicates inside the stand, at the edge of the stand and controls outside the stand of *R.* x *bohemica* at locality Mladost. Statistically significant values for p <0.05 are printed in red.

Depend.: length (cm)	Sinapis alba; Locality Mladost<br>Kruskal-Wallis test: H (2, N= 62) =12.84583 p =0.0016			
	Code	Valid N	Sum of Ranks	Mean Rank
stand	1	22	549.000	24.95455
edge	2	24	1004.000	41.83333
control	3	16	400.000	25.00000

Depend.: length (cm)	Multiple Comparisons z' values - upper right part<br>Multiple Comparisons p values (2-tailed) - lower left part		
	stand	edge	control
stand		3.169595	0.007668
edge	0.004580		2.890881
control	1.000000	0.011525	

Table S14. Results of the Kruskal-Wallis test and post-hoc comparisons of mean ranks of all pairs of groups for comparison of *Sinapis alba* seedlings Dry/Fresh mass ratio grown in pot with soil sampled in triplicates inside the stand, at the edge of the stand and controls outside the stand of *R.* x *bohemica* at locality Mladost. Statistically significant values for p <0.05 are printed in red.

Depend.: dry/fresh*100	Sinapis alba; Locality Mladost<br>Kruskal-Wallis test: H (2, N= 62) =6.610391 p =0.0367			
	Code	Valid N	Sum of Ranks	Mean Rank
stand	1	22	734.500	33.38636
edge	2	24	591.500	24.64583
control	3	16	627.000	39.18750

Depend.: dry/fresh*100	Multiple Comparisons z' values - upper right part<br>Multiple Comparisons p values (2-tailed) - lower left part		
	stand	edge	control
stand		1.641347	0.978627
edge	0.302176		2.497321
control	0.983293	0.037541	

Table S15. Results of the Kruskal-Wallis test and post-hoc comparisons of mean ranks of all pairs of groups for comparison of *Sinapis alba* seedlings Dry/Fresh mass ratio grown in pot with soil sampled in triplicates inside the stand, at the edge of the stand and controls outside the stand of *R.* x *bohemica* at locality Nasip. Statistically significant values for p <0.05 are printed in red.

Depend.: dry/fresh*100	Sinapis alba; Locality Nasip Kruskal-Wallis test: H (2, N= 58) =9.713546 p =0.0078			
	Code	Valid N	Sum of Ranks	Mean Rank
stand	1	18	603.500	33.52778
edge	2	25	840.500	33.62000
control	3	15	267.000	17.80000

Depend.: dry/fresh*100	Multiple Comparisons z' values - upper right part Multiple Comparisons p values (2-tailed) - lower left part		
	stand	edge	control
stand		0.017667	2.664053
edge	0.017667		2.868420
control	2.664053	2.868420	

Table S16. Results of the Kruskal-Wallis test and post-hoc comparisons of mean ranks of all pairs of groups for comparison of *Triticum aestivum* seedlings Dry/Fresh mass ratio grown in pot with soil sampled in triplicates inside the stand, at the edge of the stand and controls outside the stand of *R.* x *bohemica* at locality Mladost. Statistically significant values for p <0.05 are printed in red.

Depend.: dry/fresh*100	Triticum aestivum; Locality Mladost Kruskal-Wallis test: H (2, N= 67) =17.24147 p =0.0002			
	Code	Valid N	Sum of Ranks	Mean Rank
stand	1	23	500.500	21.76087
edge	2	24	850.500	35.43750
control	3	20	927.000	46.35000

Depend.: dry/fresh*100	Multiple Comparisons z' values - upper right part Multiple Comparisons p values (2-tailed) - lower left part		
	stand	edge	control
stand		2.405462	4.127496
edge	0.048456		1.849768
control	0.000110	0.193041	

Table S17. Results of the Kruskal-Wallis test and post-hoc comparisons of mean ranks of all pairs of groups for comparison of pH value of the soil sampled in triplicates at the location Mladost, inside the stand, at the edge of the stand and controls outside the stand (statistically significant values for $p < 0.05$ are printed in red).

Depend.: pH	Locality Mladost<br>Kruskal-Wallis test: H (2, N= 9) =6.488889 p =0.039			
	Code	Valid N	Sum of Ranks	Mean Rank
control	1	3	6.000	2.00000
edge	2	3	16.000	5.33333
stand	3	3	23.000	7.66667

Depend.: length (cm)	Multiple Comparisons z' values - upper right part<br>Multiple Comparisons p values (2-tailed) - lower left part		
	control	edge	stand
control		1.490712	2.534210
edge	0.408111		1.043498
stand	0.033810	0.890153	

Lab protocol LP1. Kotzmann method for humus content determination.

Humus content was determined using the Kotzmann method, through the consumption of potassium permanganate ($KMnO_4$), a strong oxidant. 0.2 g of ground soil was mixed with 130 ml of distilled water in an Erlenmeyer flask with the addition of 20 ml 32% H_2SO_4 and 50 ml of 0.1 M $KMnO_4$. The suspensions were cooked, boiling for 10 minutes, immediately after which the excess $KMnO_4$ was titrated using 0.1 M oxalic acid (H_2C2O_4) until discoloration. Since the decolorization is very slow, the excess of added oxalic acid was retitrated with 0.1 M $KMnO_4$ solution. Humus content of the samples was calculated according to the following formulas:

$$\omega\,(\mathrm{C}) = \frac{\mathrm{V}\,(\mathrm{g}/cm^3) * \,0.000514\,\mathrm{g}}{m\,(g)} * 100$$

$$\omega\,(\mathrm{humus}) = \,\omega\,(\mathrm{C}) * \,1.72$$

ω – mass fraction, V – volume of 0.1 M $KMnO_4$ spent for oxidation, 0.000514 g – mass of carbon that oxidizes 1 ml of 0.1 M $KMnO_4$ solution, m – mass of soil sample
